# A Proof-of-Concept for a Continuous-Temperature Circulating Water Bath in Frostbite Limb Rewarming

**DOI:** 10.1093/jbcr/iraf073

**Published:** 2025-05-04

**Authors:** Robert McKenzie, Zoë Anderson, Sebastian Kilcommons, Joshua N Wong, Alexis Armour

**Affiliations:** Faculty of Medicine and Dentistry, Department of Surgery, University of Alberta, Edmonton, Alberta, Canada; Faculty of Medicine and Dentistry, Department of Surgery, University of Alberta, Edmonton, Alberta, Canada; Faculty of Medicine and Dentistry, Department of Surgery, University of Alberta, Edmonton, Alberta, Canada; Faculty of Medicine and Dentistry, Department of Surgery, University of Alberta, Edmonton, Alberta, Canada; Faculty of Medicine and Dentistry, Department of Surgery, University of Alberta, Edmonton, Alberta, Canada

**Keywords:** frostbite treatment, frostbite rewarming, frostbite injury, freezing cold injury

## Abstract

Frostbite is a thermal tissue injury that can occur following prolonged exposure to freezing temperatures, often resulting in tissue ischemia and sometimes requiring amputation. The American Burn Association recommends rapid rewarming of frostbite injuries in 38–42 °C water for 15–30 minutes, but clinical application of this recommendation is often inconsistent. Our objective was to find a method to better facilitate frostbite treatment by creating a continuous-temperature circulating water bath and demonstrating its effectiveness with a proof-of-concept, descriptive study. We hypothesized that this design would effectively rewarm chilled extremities within 30 minutes without healthcare workers being required to continually monitor and maintain water temperature. We constructed a continuous-temperature circulating water bath system using a reservoir and an Anova Precision Cooker NANO. Pig feet were chilled and then immersed in 39.0 °C water with or without the Anova Precision Cooker NANO. Without the Anova Precision Cooker NANO, tissue warmed from 3.2 ± 0.3 °C to 34.2 ± 0.2 °C over 30 minutes (final water temperature of 36.5 ± 0.1 °C). With it, tissue warmed from 2.7 ± 0.2 °C to 36.7 ± 0.2 °C (final water temperature of 39.1 ± 0.1 °C). The continuous-temperature circulating water bath offers a standardized, reliable, and effective method for rewarming hypothermic tissue. Our approach could provide a solution to inconsistent and impractical frostbite rewarming methods in clinical settings to better promote rewarming compliance. Further studies are ongoing to validate the feasibility of using the continuous-temperature circulating water bath in clinical practice.

## INTRODUCTION

The incidence of frostbite (FB) is dependent on the cold temperature trends of a geographical area. In Edmonton, Alberta, Canada, where this research was conducted, winter temperatures are consistently below freezing, with the lowest recorded temperature of 2024 surpassing −46.6 °C (−50.0 °C when accounting for wind chill). Between 2010 and 2024, there have been over 15 000 recorded Emergency Department visits in Alberta involving FB, making this research relevant to the local population.

Despite the American Burn Association’s (ABA) recommendation that FB injuries receive immediate rapid rewarming of affected extremities in 38–42 °C water for 15–30 minutes, clinical delivery of this recommendation is often inconsistent.^1^ Rapid immersion rewarming is vital to increase cell viability post-injury, yet it has been reported that 35%–59% of patients received no rewarming following an FB injury.^2–4^ FB poses a logistical challenge to healthcare workers when trying to reverse tissue damage. Practical implementation of rewarming in a hospital can be hindered by multiple patients presenting within a short time frame (eg, during a “cold snap”), which can lead to staff shortage as well as space and equipment limitations. As a result, many patients do not get rewarmed per the ABA’s recommendations. For example, the current protocol for FB rewarming in Alberta involves a healthcare worker manually and continually adding warm water to a basin containing the patient’s affected limb. This process is labor-intensive and also makes it challenging to produce consistent rewarming.

We hypothesized that the continuous-temperature circulating (CTC) water bath design would effectively rewarm chilled pig feet within 30 minutes without needing to monitor or adjust the water temperature constantly. Our aim with this design was to develop and test a cost-effective, reliable, and easy-to-use rewarming device, which could 1 day be used to improve the consistent delivery of emergency treatment of acute FB.

## METHODS

The experimental CTC water bath was built using a reservoir (64.4L Cambro CamWear 182612CW), a footrest (30 cm × 50 cm wire rack), and a pump with a circulating water heater (Anova Precision Cooker NANO [APCN]). To test the effectiveness of the design, 20 fresh pig feet (approximately 1 lb per foot) were obtained, individually sealed, labeled, and placed in a refrigerator set to 3 °C for 12 hours. An initial temperature reading was taken from each pig foot along the foot’s distal phalanx just deep to the bulb (sole) of the digital cushion. Both water and tissue temperature were confirmed throughout the experiment using a ThermoPRO TP03 digital instant-read thermometer with a built-in needle probe.

Four experiments were performed, each with initial water bath temperatures of 39.0°C (Table 1). Experiments 1 and 2 were performed without chilled pig tissue, while experiments 3 and 4 were repeated with 10 different pig feet.

**Table 1.** Summary of Experiments Performed

Experiment #	Variable measured	CTC condition
1	Water temperature after 30 minutes.	No APCN.
2	Water temperature after 30 minutes.	APCN set to 39.0 °C.
3	Water temperature and pig foot tissue temperature before and after 30 minutes of immersion in 39.0 °C water.	No APCN.
4	Water temperature and pig foot tissue temperature before and after 30 minutes of immersion in 39.0 °C water.	APCN set to 39.0 °C.

Abbreviations: APCN, Anova Precision Cooker NANO; CTC, continuous-temperature circulating.

Statistical analysis was performed using the paired *t*-test with a significance level of 0.05 as the threshold for statistical significance.

## RESULTS

**Figure 1.**
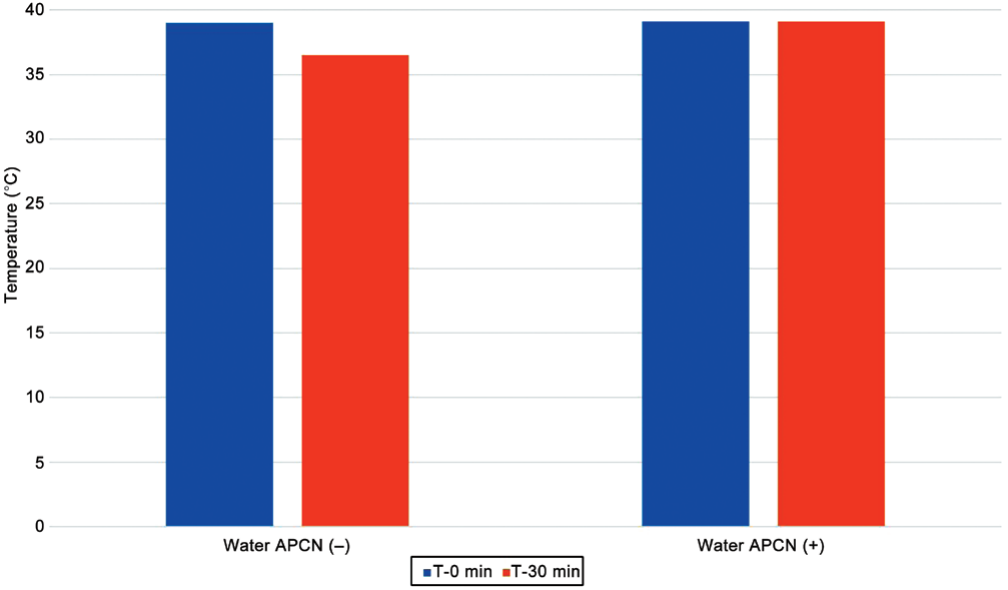
Water Temperature at T-0 and T-30 Minutes With and Without an Anova Precision Cooker Nano

A decrease in water temperature was observed after 30 minutes when no APCN was used (experiment 1). No difference in water temperature was observed when an APCN was used; the water temperature remained at 39.0 °C (experiment 2) (Figure 1). Tissue temperatures were significantly higher following 30 minutes of immersion (both experiments 3 and 4), with a greater increase observed in the group continuously warmed by the APCN (experiment 4) (Table 2)

**Table 2.** Results of Experiments 1–4

Experiment #	Results
1	The water temperature was 39.0 °C and declined to 37.0 °C over 30 minutes.
2	The water temperature was 39.0 °C and remained at 39.0 °C over 30 minutes.
3	The tissue had mean temperature at T-0 and T-30 minutes of 3.2 ± 0.3 °C and 34.2 ± 0.2°C, respectively. The mean temperature increase was 31.0 ± 0.3 °C. Final water temperature was 36.5 ± 0.1 °C
4	The tissue had mean temperature at T-0 and T-30 minutes of 2.7 ± 0.2 °C and 36.7 ± 0.2 °C, respectively. The mean temperature increase was 34.0 ± 0.2 °C. Final water temperature was 39.1 ± 0.1 °C.

**Figure 2.**
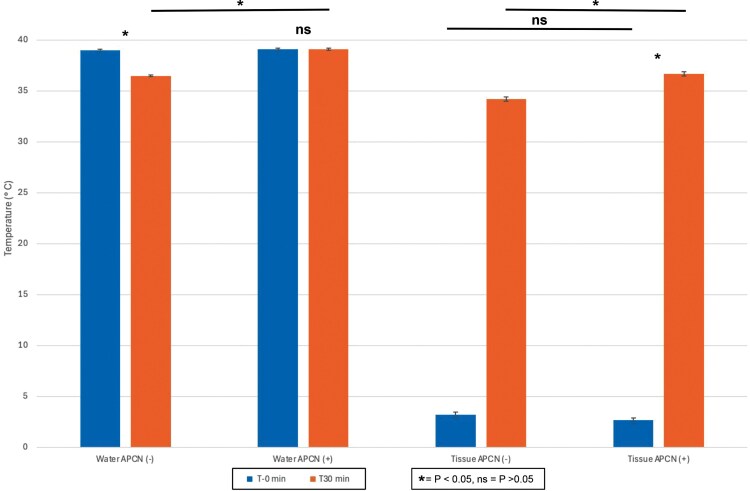
Water and Tissue Temperature at T-0 and T-30 Minutes With and Without an Anova Precision Cooker Nano

Additionally, the T-0-minute tissue temperatures of chilled pig feet tissue were not found to be statistically different, while the T-30-minute temperatures were found to be statistically greater in the APCN group (Figure 2).

## DISCUSSION

This study demonstrates that the CTC bath design was able to maintain a stable water temperature at 39.0 °C, which also offered an effective method to rewarm chilled tissue. These findings suggest that the CTC bath design could provide a practical solution to the inconsistent and impractical nature of current FB rewarming methods in clinical settings. Critical factors contributing to rewarming compliance and effectiveness are water temperature regulation and consistency.^5^ The proposed CTC bath provides a highly regulated intervention with minimal monitoring required.

This work builds on previous studies done to show the viability of CTC in maintaining a circulating warm water bath, and its potential application for medical use such as FB treatment.^6,7^ A multicentre study supports the application of a CTC water bath model in clinical practice and shows evidence of its safety, reporting zero adverse events and effective rewarming of FB injury.^8^ However, this study had a small sample size (*n* = 7, with 16 affected limbs) due to the scarcity of FB injury at the research sites. The next step and focus of our upcoming research is to compare our CTC model to current FB rewarming practices in an Edmonton, Alberta, Emergency Department with a randomized controlled design. With the prevalence of FB injury in Alberta, we anticipate being able to obtain a larger patient sample size to build on the current state of this research, as well as effectively compare the CTC model to current practices.

Our study has several limitations, mostly due to the preclinical nature of its design and that our CTC water bath has not been tested on human tissues or on a limb with FB. While the chilled pig’s feet were a viable substitute for human tissue in preclinical testing, the next step is to test it on human participants. Nonviable pig feet will rewarm without restored circulation. Although the final temperature of the tissue after rewarming was much closer to euthermia with the assistance of APCN, there is a good chance that viable limb rewarming would reach euthermia faster given the restoration of circulation. These questions could be answered in the future design involving rewarming of cold human injured limbs.

## CONCLUSION

This research demonstrates the effective rewarming of chilled tissues using a CTC water bath. Further research will be conducted to develop and test this cost-effective, reliable, and easy-to-use rewarming device in human trials to improve the consistency and delivery of acute FB treatment.
